# Lipid Profiles of College Female Student-Athletes Participating at Different Competition Levels of Organized Sport

**DOI:** 10.3389/fspor.2022.841096

**Published:** 2022-03-15

**Authors:** Kaila A. Vento, Ferdinand Delgado, Heidi Lynch

**Affiliations:** ^1^College of Health Solutions, Arizona State University, Phoenix, AZ, United States; ^2^Kinesiology Department, Point Loma Nazarene University, San Diego, CA, United States

**Keywords:** women, physical activity, sports, lipid health, cardiovascular disease, prevention, collegiate, exercise

## Abstract

College-aged women are not meeting weekly moderate-to-vigorous physical activity to support lipids protecting against cardiovascular disease onset. Participating in organized sport could assist in physical activity engagement and maintenance while positively impacting lipids predicting cardiovascular disease. However, women may be discouraged from participating in sports if they perceive benefits to be mostly seen at the higher competition levels, thus hindering seeking sports involvement at lesser-intensity levels. A total of *N* = 78 female athletes participating at the National Collegiate Athletic Association (*n* = 21), National Junior College Athletic Association (*n* = 29), and student club (*n* = 28) completed a personal characteristics questionnaire and provided blood samples to compare physical activity involvement and lipid health profiles. Linear regression modeling was used to assess how the independent variables (i.e., sport level, age, systolic blood pressure, race/ethnicity, sport/physical activity involvement hours per week, and years playing sports) on the dependent variables [i.e., total cholesterol, high density lipid (HDL), and total cholesterol-HDL ratio]. Total sample cholesterol 165.7 ± 34.0, HDL 62.8 ± 15.03, and total cholesterol-HDL ratio 2.8 ± 0.6 were all within a healthy recommendation range, along with per sport level. NJCAA sport level significantly predicted lower total cholesterol, *p* = 0.043, while identifying as Black significantly predicted higher total cholesterol, *p* = 0.008. Similarly, identifying as Black significantly predicted higher HDL, *p* = 0.021. Furthermore, increased systolic blood pressure significantly predicted higher total cholesterol-HDL ratios, *p* = 0.014. Organized sports participation may help meet physical activity requirements, support healthy lipid profiles, and ward off cardiovascular disease development in female college students regardless of competition level.

## Introduction

College-aged women in the United States of America (USA) are strongly encouraged to participate in weekly moderate-to-vigorous physical activity to support lipids protecting against cardiovascular disease (CVD) onset. CVD affects 42.1 million women over 20 years of age and is the leading cause of mortality, accounting for ~1 of every 3 female USA deaths (Garcia et al., 2016). Moderate-to-vigorous physical activity helps reduce the development of plaque buildup in the arteries by increasing high-density lipoprotein (HDL) cholesterol, supporting total cholesterol-HDL ratio be below <3.5 (i.e., dividing the total cholesterol number by the HDL cholesterol number). The American Heart Association (AHA) and American College of Sports Medicine (ACSM) recommend a minimum of 20 or more minutes of vigorous physical activity 3 or more days per week, 30 min or more of moderate physical activity 5 or more days per week, or a combination of both to boost lipid health (Pauline, 2013).

Despite the importance of physical activity's critical role in supporting lipid health, college women are not meeting weekly minutes of moderate-to-vigorous physical activity recommendations (Pauline, 2013). Pauline (2013) found 47% of college women meeting the weekly vigorous and 17.4% moderate physical activity recommendations, with 20.4% participating in neither vigorous nor moderate exercise. Compared to college-aged men, women had significantly lower exercise coping and scheduling self-efficacy (Pauline, 2013), having less confidence in their ability to engage in or plan for physical activity when confronted with challenging circumstances (i.e., tiredness and rearranging schedule). Lower exercise self-efficacy reduces minutes engaging in physical activity, contributing to poorer lipid health and increased CVD risk.

Participating in organized sport could assist in physical activity engagement and maintenance while positively impacting lipids predicting CVD. Sports can foster a sense of belonging and supportive community, encouraging women's membership and continuation (Lundvall and Walseth, 2014). Women participating in sport have better lipid health are also less likely to report having a chronic illness, including CVD, than those involved in conditioning exercise, household tasks, or recreation; differences remained after adjusting for personal demographics (i.e., age, income, education, race/ethnicity) (Scheers et al., 2008; Pharr and Lough, 2016). Furthermore, college female athletes have lower total cholesterol-HDL ratio values (Blessing et al., 1966). Within a college setting, sports organizations vary in competitiveness, including the National Collegiate Athletic Association (NCAA), National Junior College Athletic Association (NJCAA), and student club (Club) sport levels. Though less common, colleges may not have a nationally recognized athletic department or club sports yet have available intramural sports. However, it is unknown whether the competition level of organized sport contributes to heightened lipid profile health.

It is important to determine if the level of college organized sport influences lipid values when promoting women's sports participation. Women may be discouraged from participating in sports if they perceive benefits to be mostly seen at the higher competition levels, thus hindering seeking sports involvement at lesser-intensity levels. Therefore, the study's primary purpose was to examine college USA female student-athletes' lipid profiles participating at different organized sports competition levels. It was hypothesized that lipid profiles would be within a healthy range and comparable across women participating in NCAA, NJCAA, and Club sports. We expected all women participating in sports, regardless of competition level, to meet physical activity weekly minute requirements, leading to similar lipid profile health values.

## Methods

### Design

The study was cross-sectional, entailing one 45 min visit to a designated space at the participating university and community college's athletic facilities. Participants' height, weight, blood pressure, and lipid profile were assessed, followed by one questionnaire administered for personal demographics via the web-based platform Qualtrics during the hours between 7:00 and 10:00 A.M. Before study participation, informed consent was given in writing by all participants. The study was approved by the Arizona State University (ASUIRB, study number 00009976) and the Maricopa County Community College (MCCCIRB, study number 201904696) Institutional Review Boards. All participants received a $50.00 electronic gift card for their participation.

### Participants

A total of *N* = 78 (NCAA = 21; NJCAA = 29; Club = 28) provided blood samples. Participants were eligible if they were a female athlete at least 18 years old and participating in a registered collegiate or club sport at a 2- or 4-year college. Recruitment and data collection occurred between July-November of 2019 in Phoenix, AZ, USA. Study flyers and authorized athletic personnel sent an email to all female athletes and their coaches/Club presidents, providing a brief description of the study and an invitation to review the informed consent.

### Instruments

#### Personal Demographics

Age, race/ethnicity, competition level, sport/physical activity involvement hours per week, sport played, and years playing the sport were gathered. Weight (kg) was to be consistent with blood pressure, derived from a mobile device to provide an instant measurement (Omron Mobile BIA, Kyoto, Japan) and height (cm) with a mobile stadiometer (SECA 213 portable stadiometer, Hamburg, Deutschland). A single resting blood pressure measurement (mmHg) was conducted with participants seated and resting quietly for 2 min before measurement (Omron 907 portable automatic monitor, Kyoto, Japan).

#### Lipid Profiles

Participants abstained from food and drink for a minimum of 12 h overnight. The following morning, fasted lipid levels of interest (mg/dl) (i.e., total cholesterol, high-density lipoprotein [HDL], and total cholesterol-HDL ratio) were collected between 7:00 and 10:00 A.M. A finger prick was utilized to draw blood via a straw and blood was transferred into a cassette and fed to a portable device to read lipid profiles within 8 min (CHOLESTECH LDX, Abbot, Hayward, CA, USA). The defined healthy value ranges per lipid variable can be seen in Table 1.

**Table 1:** Female college athletes' demographics and lipid profiles by level of sport participation.

	**Total (*N* = 78)**	**NCAA (*n* = 21)**	**NJCAA (*n* = 29)**	**Club (*n* = 28)**
**Demographics**				
Age^a^	19.7 ± 1.2	20.2 ± 1.4	18.8 ± 0.7^**^	20.1 ± 0.9
Height (cm)	166.1 ± 7.1	169.9 ± 7.1	164.9 ± 6.7	164.4 ± 6.5
Weight (kg)	65.7 ± 11.8	65.8 ± 11.7	67.5 ± 12.2	63.8 ± 11.6
BMI	23.6 ± 4.5	22.7 ± 2.9	24.3 ± 3.6	23.5 ± 3.8
Systolic blood pressure (mmHg)^b^ <120	113.5 ± 10.4	112.1 ± 7.5	117.7 ± 9.7^**^	110.1 ± 11.7
Diastolic blood pressure (mmHg) <80	73.2 ± 9.3	71.1 ± 5.7	75.2 ± 8.6	72.6 ± 11.6
Sport/physical activity hours per week^c^	13.6 ± 7.2	14.6 ± 5.8	15.9 ± 8.4	10.8 ± 6.2^*^
Sport years^d^	8.2 ± 4.9	9.9 ± 3.7	9.5 ± 4.2	6.0 ± 5.6^*^
**Race/ethnicity**	**% (** ***n*** **)**			
Black	8 (6)	0 (0)	21 (6)	0 (0)
Hispanic	15 (12)	14 (3)	21 (6)	11 (3)
White	62 (48)	86 (18)	35 (10)	71 (20)
Asian/Pacific Islander	6 (5)	0 (0)	3 (1)	14 (4)
Native American/Indian	3 (2)	0 (0)	7 (2)	0 (0)
Other	1 (1)	0 (0)	0 (0)	4 (1)
Missing	5 (4)	0 (0)	14 (4)	0 (0)
**Sport participation**	**% (** ***n*** **)**			
Basketball	5 (4)	5 (1)	10 (3)	0 (0)
Cross Country	5 (4)	14 (3)	3 (1)	0 (0)
Dance	8 (6)	0 (0)	0 (0)	21 (6)
Dragon boat	1 (1)	0 (0)	0 (0)	4 (1)
Hockey	3 (2)	0 (0)	0 (0)	7 (2)
Lacrosse	5 (4)	14 (3)	0 (0)	4 (1)
Marching band	1 (1)	5 (1)	0 (0)	0 (0)
Quidditch	4 (3)	0 (0)	0 (0)	11(3)
Rugby	5 (4)	0 (0)	0 (0)	14 (4)
Sailing	1 (1)	0 (0)	0 (0)	4 (1)
Soccer	17 (13)	0 (0)	21 (6)	25 (7)
Softball	10 (8)	0 (0)	24 (7)	4 (1)
Swimming	3 (2)	10 (2)	0 (0)	0 (0)
Track and field	18 (14)	29 (6)	28 (8)	0 (0)
Triathlon	5 (4)	10 (2)	0 (0)	7 (2)
Ultimate frisbee	1 (1)	5 (1)	0 (0)	0 (0)
Volleyball	6 (5)	5 (1)	14 (4)	0 (0)
Water polo	1 (1)	5 (1)	0 (0)	0 (0)
**Lipid profile**	**Mean** **±SD**			
Total cholesterol (mg/dL)^e^ <200	165.7 ± 34.0	170.9 ± 31.9	160.5 ± 39.6^*^	167.4 ± 29.5
HDL (mg/dL) >40 (>60 is optimal)	62.8 ± 15.0	64.1 ± 15.7	62.6 ± 14.9	62.0 ± 15.3
Total cholesterol-HDL ratio <3.5	2.8 ± 0.6	2.7 ± 0.6	2.8 ± 0.6	2.8 ± 0.7

*Values based on fasted morning samples. Sport practice sessions were not controlled. All numbers rounded to the nearest tenth and whole percentage*.

*Recommended reference for blood pressure and lipid values given*.

a *NJCAA significantly differed from Club, P < 0.001; NJCAA significantly differed from NCAA, P ≤ 0.01*.

b *NJCAA significantly differed from NCAA, P < 0.001; NJCAA significantly differed from Club, P = 0.01*.

c *Club significantly differed from NJCAA, P < 0.05*.

d *Club significantly differed from NCAA and NJCAA, P < 0.05*.

e *NJCAA significantly differed from NCAA, P ≤ 0.05*.

* *P < 0.05*.

** *P ≤ 0.01*.

### Data Analysis

For statistical analyses, the Statistical Package for Social Sciences (SPSS) Version 27 was used. Personal demographics and lipid profile values are given as frequencies (*n*), percentages (%), and mean ± standard deviation. The analysis of variances (ANOVA) tests determined sport level differences regarding personal demographic variables. Separate linear regression models analyzed the independent variables (i.e., sport level, age, systolic blood pressure, race/ethnicity, sport/physical activity hours per week, and years playing sports) on the dependent variables (i.e., total cholesterol, HDL, and total cholesterol-HDL ratio). All statistical analyses were performed with the significance level set at *p* < 0.05.

## Results

### Personal Demographics

Participants (age, 19.7 ± 1.2; height cm, 166.1 ± 7.1; weight kg, 65.7 ± 11.8; race/ethnicity, 62% White) were involved with sport/physical activities for an average of 13.6 ± 7.2 h per week. The top three participating sports in this study were track and field (*n* = 14), soccer (*n* = 13), and softball (*n* = 10), and all played their respective sports for 8.2 ± 4.9 years. Age, sport/physical activity involvement hours per week, and years playing sports differed between the sport levels, *p* < 0.05. NCJAA athletes were younger than NCAA and Club athletes. In addition, club athletes had fewer sport/physical activity hours per week than NJCAA and NCAA athletes and played fewer years of organized sport. Regarding blood pressure, NJCAA athletes (117.7 ± 9.7 mmHg) had significantly higher systolic blood pressure (*p* ≤ 0.01) compared to NCAA (112.1 ± 7.5) and Club (110.1 ± 11.7) athletes. Additional total and sport-level demographic information and *p*-values can be seen in Table 1.

### Lipid Profiles

The mean ± standard deviation for lipid values for the total sample are as follows: total cholesterol 165.7 ± 34.0 mg/dL, HDL 62.8 ± 15.03 mg/dL, and total cholesterol-HDL ratio 2.8 ± 0.6. Participants' lipid variable values were not within the defined healthy range for the following: *n* = 12 total cholesterol >200 mg/dL, *n* = 2 HDL < 40 mg/dL, and *n* = 7 total cholesterol-HDL ratio ≥3.5. The mean ± standard deviation for the lipid profile values per sport level can be seen in Table 1. Female athletes participating at the NJCAA sport level significantly predicted lower total cholesterol, *p* = 0.043, while identifying as Black significantly predicted higher total cholesterol, *p* = 0.008. Similarly, identifying as Black significantly predicted higher HDL, *p* = 0.021. Furthermore, increased systolic blood pressure significantly predicted higher total cholesterol-HDL ratios, *p* = 0.014. No other dependent variables were found to predict total cholesterol, HDL, or total cholesterol-HDL ratios. Table 2 displays the beta (SE), 95% confidence intervals (95% CI), and *p*-values for each dependent variable per model (i.e., total cholesterol, HDL, and total cholesterol-HDL ratio), along with the variance explained (*R*^2^).

**Table 2:** Linear regression model of lipid values.

	**Beta (SE)**	**95% CI**	***p*-value**	***R^**2**^***
**Total cholesterol**				
**Sport level**				
NJCAA^a^	−0.378	(−46.363, −0.807)	0.043^*^	
Club	−0.102	(−25.288, 12.854)	0.517	
Age	−0.120	(−9.600, 3.786)	0.389	
Systolic blood pressure	0.145	(−0.306, 1.128)	0.256	
**Race/Ethnicity**				0.199
Black^b^	0.397	(11.796, 74.080)	0.008^*^	
Asian/Pacific Islander	−0.086	(−38.818, 18.480)	0.481	
Native American/Indian	−0.096	(−63.945, 28.784)	0.451	
Hispanic	0.091	(−12.036, 26.116)	0.463	
Other	0.102	(−33.477, 85.473)	0.386	
Sport/physical activity hours per week	−0.194	(−1.894, 0.291)	0.147	
Sport years	−0.033	(−1.759, 1.357)	0.797	
**HDL**				
**Sport level**				
NJCAA	−0.280	(−19.961, 2.712)	0.133	
Club	−0.255	(−17.584, 2.116)	0.121	
Age	−0.151	(−5.486, 1.674)	0.291	
Systolic blood pressure	−0.189	(−0.637, 0.098)	0.148	
**Race/Ethnicity**				0.207
Black^c^	0.349	(2.934, 33.983)	0.021^*^	
Asian/Pacific Islander	0.154	(−5.502, 23.158)	0.223	
Native American/Indian	0.127	(−11.891, 34.481)	0.334	
Hispanic	0.134	(−4.471, 14.690)	0.290	
Other	0.163	(−9.267, 49.481)	0.174	
Sport/physical activity hours per week	−0.252	(−1.063, 0.036)	0.067	
Sport years	−0.122	(−1.143, 0.417)	0.356	
**Total cholesterol-HDL ratio**				
**Sport level**				
NJCAA	0.017	(−0.467, 0.511)	0.929	
Club	0.223	(−0.138, 0.712)	0.182	
Age	0.002	(−0.153, 0.155)	0.990	
Systolic blood pressure^d^	0.333	(0.004, 0.036)	0.014^*^	
**Race/Ethnicity**				0.179
Black	−0.061	(−0.807, 0.532)	0.682	
Asian/Pacific Islander	−0.249	(−1.223, 0.013)	0.055	
Native American/Indian	−0.040	(−1.149, 0.851)	0.766	
Hispanic	−0.051	(−0.495, 0.331)	0.693	
Other	−0.100	(−1.806, 0.749)	0.411	
Sport/physical activity hours per week	0.077	(−0.017, 0.030)	0.577	
Sports years	0.041	(−0.029, 0.039)	0.761	

*Values based on fasted morning samples. Sport practice sessions were not controlled. NCAA and White references values for sport level and race/ethnicity*.

a *NJCAA significantly predicted total cholesterol, P < 0.05*.

b *Black race/ethnicity significantly predicted total cholesterol, P < 0.05*.

c *Black race/ethnicity significantly predicted HDL, P < 0.05*.

d *Systolic blood pressure significantly predicted total cholesterol-HDL ratio, P < 0.05*.

* *P < 0.05*.

## Discussion

This study examined college female student-athletes' lipid profiles participating at different organized sports competition levels. Surprisingly, the sports organization's competition level did predict total cholesterol. Though total and sport level mean values for all lipids were well within the healthy reference ranges, few athletes (*n* = 12) were not within the defined ranges. Additionally, sports and training practice sessions were not controlled, possibly impacting total cholesterol levels. Yet, the mean lipid values reported from the current study are like those found by Scheers et al. (2008) in young women partaking in sports and Blessing et al. (1966) among a representative NCAA female collegiate population. Plausible behavioral mechanisms that facilitate these levels of blood lipid profiles may include as a collective the number of years playing sports, sport/physical activity hours per week, and the type of sport played. Despite hours of weekly physical activity and years of playing sports being significantly different, each sport level engaged in over 10 h of sport and non-sport-specific physical activities per week and participated in sports for at least 6 years. Therefore, regardless of level, participating on a sports team may help women meet AHA and ACSM physical activity requirements for an extended amount of time.

Race, specifically Black female athletes, significantly predicted higher total cholesterol and HDL values and systolic blood pressure with a higher total cholesterol-HDL ratio (Scheers et al., 2008; Garcia et al., 2016). While higher HDL levels are typically cardioprotective, the increase in HDL was offset by the greater increase in total cholesterol, resulting in a less favorable total cholesterol: HDL ratio (Arsenault et al., 2009). Cholesterol levels and blood pressure vary by race, ethnicity, and sex (CDC, 2017). However, according to the U.S. Centers for Disease Control and Prevention (CDC), 48% of Black women suffer from CVD and have one of the highest stroke-related deaths rates compared to other races and ethnicities (CDC, 2017). While the underlying mechanisms influencing these differences are unknown, it could be critical to target preventative measures early among Black women susceptible to CVD by increasing their physical activity levels through sports participation. These results highlight the need, even among young athletes, to monitor biomarkers for CVD, particularly among populations more at risk for CVD. Such assessments should be included in routine pre-season physical exams, for example.

The AHA recommends screening at age 21 to identify CVD risk for women and initiate intervention via lifestyle modifications (Fay et al., 2019; Hosseinzadeh and Wild, 2021). In the current study sample, athletes' ages ranged between 18 and 22 years, and thus, some of the healthy young athletes are the age that the AHA wants screenings to begin. Obstetricians/gynecologists often serve as women's primary care providers and should initiate lipid panel screenings at wellness visits. To further elucidate the impact of sport participation on lipid health of collegiate students, physical activity and exercise researchers are encouraged to examine the effectiveness of organized sports participation on lipid health in sedentary college students, comparing groups in exercise programs. To increase women's interest in sports participation at the lower competition levels to improve cardiovascular health, college sports organizations can offer free or low-cost health screenings to test blood pressure, lipid values, and recreational memberships. In addition, facilities could host an hourly weekly event, such as Women Wellness Wednesdays, setting aside sporting courts and equipment and providing information on the importance of sports participation and CVD, creating a space for women to feel comfortable engaging in sports (Fisher et al., 2018). Colleges could also offer one-credit hour for registering in a sports-activity course.

The current study promotes the continuation of organized sports participation of women within a college environment to meet physical activity requirements, maintain a healthy lifestyle, and reduce CVD onset. Strengths of this study include the sports variety among three organized competitive sports levels and physiological outcomes to complement self-reported data. Yet, it is not without its limitations. The sample did not recruit female athletes competing at the National Association of Intercollegiate Athletics (NAIA) or intramural sports levels, nor across all athletic divisions (I-III). Though women not meeting physical activity hour requirements were omitted, several studies have supported significant lipid health improvement of sedentary female students participating in various 8-week aerobic programs, including steps, dance, Zumba, and running (Kin Isler et al., 2001; Juhas et al., 2020; Turgut and Soylu, 2021). Yet, none of these studies examined sports specifically (Kin Isler et al., 2001; Juhas et al., 2020; Turgut and Soylu, 2021). In addition, the sample is small and gathered from Phoenix, AZ, USA. Future works should include a larger representative sample of organized sports competition levels across the USA colleges and universities and those reporting inactivity for better comparisons. Finally, those physically active generally have better lifestyle habits, including better eating habits, sleep, and stress management. However, it was found not to be the case for this specific group, and their overall quality of life outcomes are reported by Vento et al. (2022).

## Conclusions

Regardless of competition level, organized sports participation may help meet physical activity requirements to foster a physically active lifestyle, support healthy lipid profiles, and ward off CVD development in female college students. Women attending college are encouraged to become involved in organized sports, at least at the Club and possible intramural level. College athletic and club sport departments or recreation facilities should increase innovative promotional efforts to reach a sizeable and diverse women-student audience and supplement athletic equipment and space availability to accommodate sports membership.

## Data Availability Statement

The raw data supporting the conclusions of this article will be made available by the authors, without undue reservation.

## Ethics Statement

The study was approved by the Arizona State University (ASUIRB, study number 00009976) and the Maricopa County Community College (MCCCIRB, study number 201904696) Institutional Review Boards. The patients/participants provided their written informed consent to participate in this study.

## Author Contributions

HL designed the study. KV collected the data. KV and FD analyzed the data and undertook data interpretation. KV, FD, and HL prepared the manuscript. All authors approved the final version of the manuscript.

## Funding

The study was funded by the Global Sport Institute at Arizona State University.

## Conflict of Interest

The authors declare that the research was conducted in the absence of any commercial or financial relationships that could be construed as a potential conflict of interest.

## Publisher's Note

All claims expressed in this article are solely those of the authors and do not necessarily represent those of their affiliated organizations, or those of the publisher, the editors and the reviewers. Any product that may be evaluated in this article, or claim that may be made by its manufacturer, is not guaranteed or endorsed by the publisher.
